# Bioactive Phytochemicals Isolated from *Akebia quinata* Enhances Glucose-Stimulated Insulin Secretion by Inducing PDX-1

**DOI:** 10.3390/plants9091087

**Published:** 2020-08-24

**Authors:** Dahae Lee, Jin Su Lee, Jurdas Sezirahiga, Hak Cheol Kwon, Dae Sik Jang, Ki Sung Kang

**Affiliations:** 1 College of Korean Medicine, Gachon University, Seongnam 13120, Korea; pjsldh@naver.com; 2 Department of Life and Nanopharmaceutical Sciences, Graduate School, Kyung Hee University, Seoul 02447, Korea; lee2649318@naver.com; 3 College of Medicine and Health Sciences, University of Rwanda, Kigali 3286, Rwanda; jurdas30@gmail.com; 4 Natural Products Research Center, Korea Institute of Science and Technology, Gangneung 210-340, Korea; hkwon@kist.re.kr

**Keywords:** *Akebia quinata*, stigmasterol-3-*O*-β-d-glucoside, insulin secretion, IRS-2, PI3K, Akt, PDX-1

## Abstract

Chocolate vine (*Akebia quinata*) is consumed as a fruit and is also used in traditional medicine. In order to identify the bioactive components of *A. quinata*, a phytosterol glucoside stigmasterol-3-*O*-β-d-glucoside (1), three triterpenoids maslinic acid (2), scutellaric acid (3), and hederagenin (4), and three triterpenoidal saponins akebia saponin PA (5), hederacoside C (6), and hederacolchiside F (7) were isolated from a 70% EtOH extract of the fruits of *A. quinata* (AKQU). The chemical structures of isolates 1–7 were determined by analyzing the 1D and 2D nuclear magnetic resonance (NMR) spectroscopic data. Here, we evaluated the effects of AKQU and compounds 1–7 on insulin secretion using the INS-1 rat pancreatic β-cell line. Glucose-stimulated insulin secretion (GSIS) was evaluated in INS-1 cells using the GSIS assay. The expression levels of the proteins related to pancreatic β-cell function were detected by Western blotting. Among the isolates, stigmasterol-3-*O*-β-d-glucoside (1) exhibited strong GSIS activity and triggered the overexpression of pancreas/duodenum homeobox protein-1 (PDX-1), which is implicated in the regulation of pancreatic β-cell survival and function. Moreover, isolate 1 markedly induced the expression of extracellular signal-regulated protein kinases 1 and 2 (ERK1/2), insulin receptor substrate-2 (IRS-2), phosphoinositide 3-kinase (PI3K), and Akt, which regulate the transcription of PDX-1. The results of our experimental studies indicated that stigmasterol-3-*O*-β-d-glucoside (1) isolated from the fruits of *A. quinata* can potentially enhance insulin secretion, and might alleviate the reduction in GSIS during the development of T2DM.

## 1. Introduction

Diabetes mellitus (DM) is one of the top 10 causes of global deaths, and affected 463 million people worldwide in 2019. Experts expect that the number of patients with DM will increase to 578 million by 2030 [1,2]. Type 2 diabetes mellitus (T2DM) is a metabolic disease, which comprises approximately 90% of all cases of DM [3]. The clinical characteristics of patients with T2DM are decreased insulin production and impaired insulin function, which leads to persistent hyperglycemia [4]. Patients with T2DM are unable to control the disease by lifestyle changes alone, and require drugs and insulin for reducing the hyperglycemia. Therefore, one of the major factors in the development of T2DM is the reduction of glucose-stimulated insulin secretion (GSIS) in pancreatic β-cells [5]. Numerous therapeutic strategies have been developed at present, however, only a handful of patients with T2DM achieve long-term glycemic control [6]. The first generation sulfonylureas continually induce the secretion of insulin regardless of the blood glucose levels, which consequently results in hypoglycemia [7]. Apart from the sulfonylurea class of drugs, the current oral anti-diabetic drugs, including dipeptidyl peptidase-4 (DPP-4) inhibitors, α-glucosidase inhibitors, biguanides, and thiazolidinediones, have been reported to have numerous side effects [8]. DPP-4 inhibitors reduce the postprandial glucose levels by inhibiting DPP-4, and have numerous side effects, including hypersensitivity, nasopharyngitis, and headache [9]. The α-glucosidase inhibitors delay the digestion and absorption of carbohydrates, which suppresses postprandial hyperglycemia, while the biguanide drugs increase insulin sensitivity. The latter two classes of drugs have been found to be associated with gastrointestinal (GI) side effects. [10]. Thiazolidinediones are peroxisome proliferators-activated receptor γ (PPAR-γ) agonists, and they improve insulin function while simultaneously causing hepatotoxicity [11]. Therefore, efforts to develop safer and more effective anti-diabetic drugs remain an important challenge.

In step with the trend, numerous studies have been increasingly exploring the utilization of natural products in the development of anti-diabetic drugs in the recent years. Numerous plant-derived compounds can directly enhance insulin secretion both in vitro and in vivo, and have attracted attention as sources of novel pharmaceuticals [12,13,14,15]. Although these plant-derived compounds are still not clinically available, such studies are significant as they provide a reference for the identification of novel molecules or pathways that have a potential in controlling hyperglycemia. The common metabolic targets for natural products with anti-diabetic activity are largely divided into three. The first category of natural products target and control sugar homeostasis, the second category enhances insulin synthesis and secretion, while the third category regulates the insulin signaling pathway [10]. In T2DM, the dysfunction of pancreatic β-cells delay the rate of insulin secretion and could be indicative of disease progression [16]. Although the exact mechanism remains unclear, pancreas/duodenum homeobox protein-1 (PDX-1) and V-maf musculoaponeurotic fibrosarcoma oncogene homolog A (MAFA) are implicated in the development of pancreatic β-cells and the transcription of the insulin gene [17,18]. Defects in the glucose-stimulated transcription of the insulin gene in a *Psammomys obesus* model of T2DM resulted in 80–90% lower insulin secretion than that associated with PDX-1 deficiency, whereas MAFA remained unaffected [19]. This indicates that PDX-1 is essential for the glucose-stimulated transcription of the insulin gene. Therefore, identification of novel natural products that improve GSIS and enhance the expression of PDX-1 may be beneficial for the development of safer and more effective anti-diabetic drugs.

*Akebia quinata* (Thunb.) Decne. is a creeping woody vine that is distributed in East Asia, including Korea, China, and Japan [20,21,22]. It belongs to the Lardizabalaceae family of plants and is commonly known as “wild banana” in China and “Chocolate vine” in the Unites States [21,22,23]. Owing to their pharmacological activities, the fruits of *A. quinata* are generally used in Traditional Chinese Medicine as antiphlogistic [24], antineoplastic, and antidiuretic agents [23,24]. However, to the best of our knowledge, no previous studies have investigated the anti-diabetic activity of *A. quinata* and the compounds isolated from *A. quinata*. In our efforts to identify the bioactive compounds of *A. quinata* that have anti-diabetic properties, we performed a chemical investigation of a 70% EtOH extract of the fruits of *A. quinata* (AKQU). The effects of AKQU and the isolated compounds on insulin secretion were additionally evaluated using this GSIS assay, and the GSIS activity was expressed in terms of the glucose stimulated index (GSI). GSI reflected the GSIS ratio of INS-1 cells stimulated by 16.7 and 3.3 mM glucose. Western blotting was performed for investigating the mechanisms underlying the observed activity in INS-1 cells. The INS-1 rat pancreatic β-cell line secretes insulin in response to concentrations of glucose [25].

**Figure 1:**
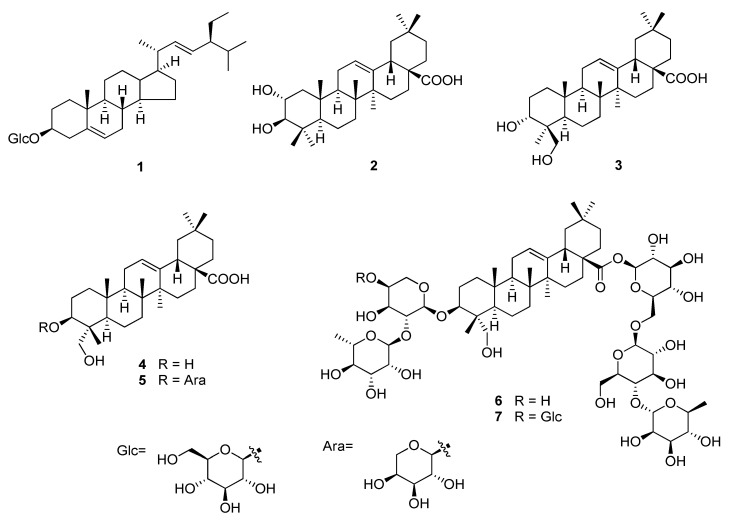
Chemical structures of compounds 1–7.

In this study, we found that the AKQU increased GSIS in INS-1 cells. The chemical analysis of AKQU led to the isolation of a phytosterol glucoside (1), three triterpenoids (2–4), and three triterpenoidal saponins (5–7). The chemical structures of the isolates 1–7 were determined by interpretation of the 1D and 2D nuclear magnetic resonance (NMR) spectroscopic data (Figure 1). From the GSIS assay, we identified that compounds 1–5 induced insulin secretion in INS-1 cells. Interestingly, the effect of compound 1 on insulin secretion was similar to that of the oral sulfonylurea drug, gliclazide, that induces hypoglycemia, at a concentration of 10 µM. Additionally, only compound 1 induced the overexpression of PDX-1. Compound 1 also induced the overexpression of extracellular signal-regulated protein kinases 1 and 2 (ERK1/2), insulin receptor substrate-2 (IRS-2), phosphoinositide 3-kinase (PI3K), and Akt, which are associated with β-cell function and enhanced insulin secretion, via mechanisms upstream of PDX-1. Herein, we report the isolation of compounds 1–7, along with their effects on insulin secretion and the underlying mechanisms of action.

## 2. Results

### 2.1. Identification of Compounds 1–7

Seven compounds (1–7) were isolated from the fruits of *A. quinata* in this study. The compounds were identified as stigmasterol-3-*O*-β-d-glucoside (1) [26], maslinic acid (2) [27], scutellaric acid (4-epihederagenin) (3) [28], hederagenin (4) [29], akebia saponin PA (5) [28], hederacoside C (kalopanax-saponin B) (6) [30], and hederacolchiside F (7) [31] by analysis of the 1D and 2D NMR spectroscopic data and by comparison with published values (Figure 1).

### 2.2. Effects of AKQU and Compounds 1–7 on GSIS

**Figure 2:**
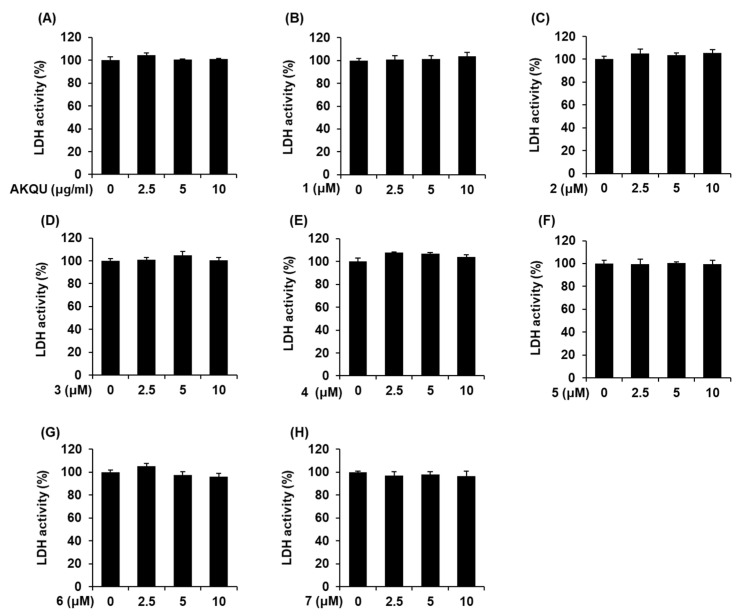
Effect of *A. quinata* (AKQU) and compounds 1–7 on the lactate dehydrogenase (LDH) activity in INS-1 cells. Effect of (**A**) AKQU, (**B**–**H**) compounds 1–7 on the LDH activity in INS-1 cells following 24 h of treatment, compared with that of the control (0 μg/mL or 0 μM), as determined by the LDH release assay (*n* = 3 independent experiments, *p* > 0.05, Kruskal–Wallis non-parametric test). The data are presented as the mean ± SEM.

**Figure 3:**
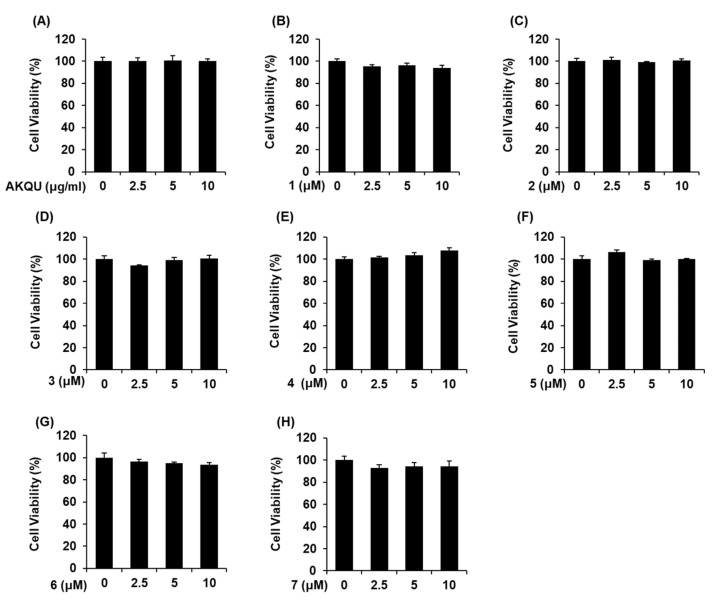
Effect of AKQU and compounds 1–7 on the viability of INS-1 cells. Effect of (**A**) AKQU, (**B**–**H**) compounds 1–7 on the viability of INS-1 cells following 24 h of treatment, compared with that of the control (0 μg/mL or 0 μM), as determined by the MTT assay (*n* = 3 independent experiments, *p* > 0.05, Kruskal–Wallis non-parametric test). The data are presented as the mean ± SEM.

**Figure 4:**
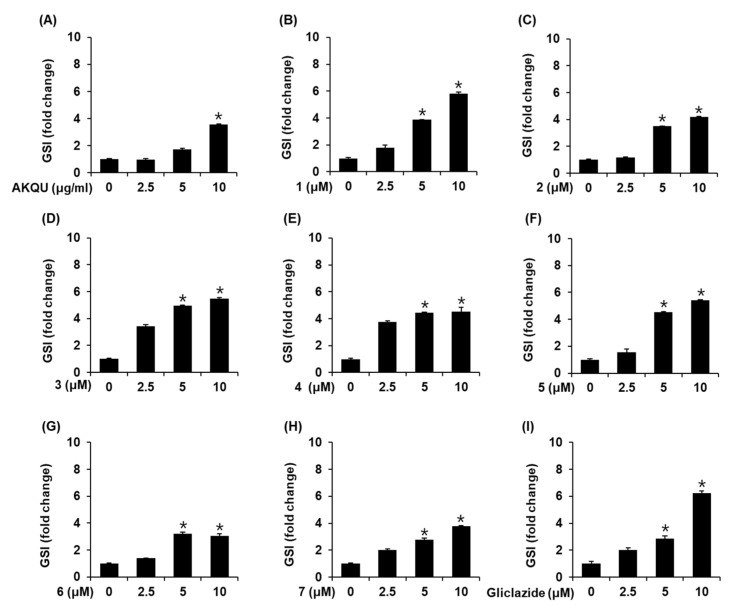
Effect of AKQU and compounds 1–7 on the glucose-stimulated insulin secretion (GSIS) in INS-1 cells. Effect of (**A**) AKQU, (**B**–**H**) compounds 1–7, and (**I**) gliclazide (positive control) on the GSIS in INS-1 cells following 1 h of treatment, compared with that of the control (0 μg/mL or 0 μM), as determined by the insulin secretion assay. GSIS was expressed in terms of the glucose stimulated index (GSI). (*n* = 3 independent experiments, * *p* < 0.05 compared with control, Kruskal–Wallis non-parametric test). The data are presented as the mean ± SEM.

In order to determine the non-toxic concentrations of AKQU and compounds 1–7, we performed Ez-Cytox cell viability and lactate dehydrogenase (LDH) release assay using INS-1 cells. As depicted in Figure 2 and Figure 3, AKQU and compounds 1–7 showed no toxicity at all concentrations. As depicted in Figure 4A, AKQU increased the GSIS in a concentration-dependent manner. The values of GSI were 0.98 ± 0.05, 1.73 ± 0.06, and 3.58 ± 0.01 at concentrations of 2.5, 5, and 10 μg/mL, respectively. These results indicated that AKQU stimulates insulin secretion in INS-1 cells without inducing cytotoxicity. As depicted in Figure 4, compounds 1–7 increased the GSI at concentrations of 2.5, 5, and 10 μM. Among these, compounds 1–5 increased the GSI by more than four fold. The values of GSI were 5.82 ± 0.10, 4.20 ± 0.01, 5.45 ± 0.08, 4.50 ± 0.34, and 5.41 ± 0.02 for compounds 1, 2, 3, 4, and 5, respectively, at a concentration of 10 μM. Among these five compounds, the GSI of stigmasterol-3-*O*-d-glucoside (1) was the highest, and its GSIS activity increased in a concentration-dependent manner (Figure 4B). The GSI of stigmasterol-3-*O*-d-glucoside (1) was similar to that of the same concentration of gliclazide (Figure 4I). The GSI of 10 μM gliclazide was 6.22 ± 0.16. The insulin secretion assays revealed that compounds 1–5 isolated from *A. quinata* stimulated insulin secretion in INS-1 cells without inducing cytotoxicity. We therefore performed further mechanistic studies with compounds 1–5.

### 2.3. Effect of AKQU and Compounds **1**–**5** on the Expression of PDX-1 Protein

**Figure 5:**
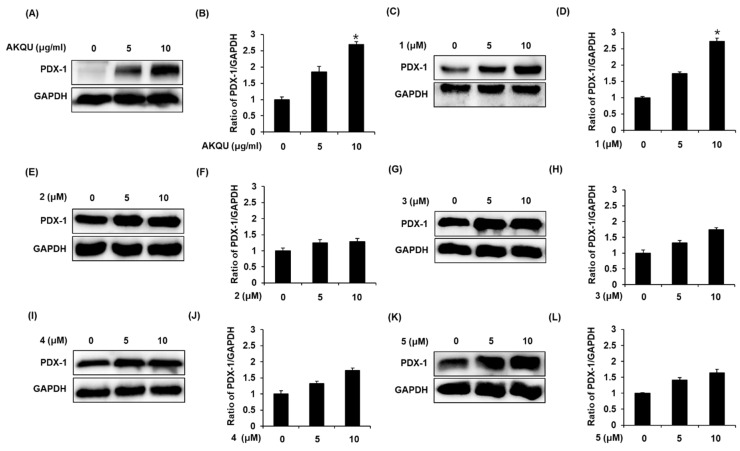
Effect of AKQU and compounds 1–5 on the expression levels of pancreas/duodenum homeobox protein-1 (PDX-1) protein in INS-1 cells. (**A**,**C**,**E**,**G**,**I**,**K**) Expression levels of PDX-1 and GAPDH proteins in INS-1 cells treated or untreated with AKQU (5 and 10 μg/mL) and compounds 1–5 (5 and 10 μM) for 24 h. (**B**,**D**,**F**,**H**,**J**,**L**) The bar graphs represent the densitometric quantification of the bands obtained by Western blotting (*n* = 3 independent experiments, * *p* < 0.05 compared with control, Kruskal–Wallis non-parametric test).

In order to investigate the underlying molecular mechanisms by which AKQU and compounds 1–5 affected insulin secretion, Western blotting was performed for quantifying the expression of PDX-1, which is involved in pancreatic β-cell function. As depicted in Figure 5A,C, the expression levels of PDX-1 protein markedly increased following treatment with AKQU and stigmasterol-3-*O*-β-d-glucoside (1) in a concentration-dependent manner. The bar graphs depict the ratio of PDX-1 expression normalized to that of GAPDH (Figure 5B,D). The other compounds did not induce any changes in the expression of PDX-1 protein.

### 2.4. Effect of Stigmasterol-3-O-β-d-Glucoside (1) on the Protein Expression of P-ERK1/2, ERK1/2, P-IRS-2, IRS-2, P-PI3K, PI3K, P-Akt (Ser473), and Akt

**Figure 6:**
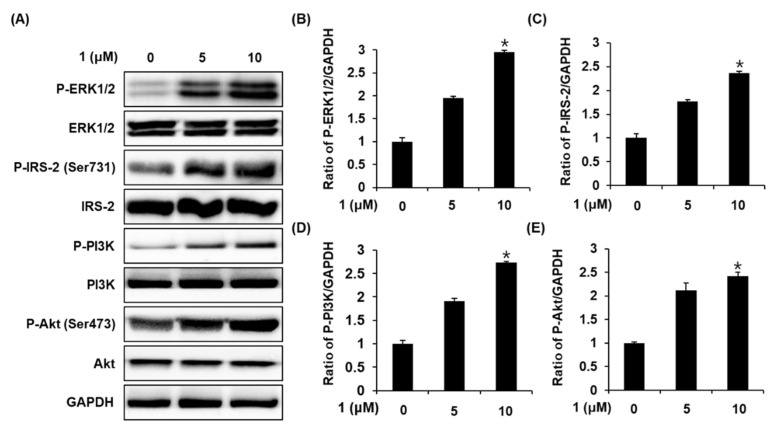
Effect of stigmasterol-3-*O*-β-d-glucoside (1) on the expression levels of phospho-extracellular signal-regulated protein kinases 1 and 2 (ERK1/2), ERK1/2, phospho-insulin receptor substrate-2 (P-IRS-2), (Ser731), IRS-2, phospho-phosphoinositide 3-kinase (P-PI3K), PI3K, phospho-Akt (P-Akt) (Ser473), and Akt proteins in INS-1 cells. (**A**) The expression levels of P-ERK1/2, ERK1/2, P-IRS-2 (Ser731), IRS-2, P-PI3K, PI3K, P-Akt (Ser473), Akt, and GAPDH proteins in INS-1 cells treated or untreated with stigmasterol-3-*O*-β-d-glucoside (1) at concentrations of 5 and 10 μM for 24 h. (**B**–**E**) The bar graphs represent the densitometric quantification of the bands obtained by Western blotting (*n* = 3 independent experiments, * *p* < 0.05 compared with control, Kruskal–Wallis non-parametric test). The data are presented as the mean ± SEM.

**Figure 7:**
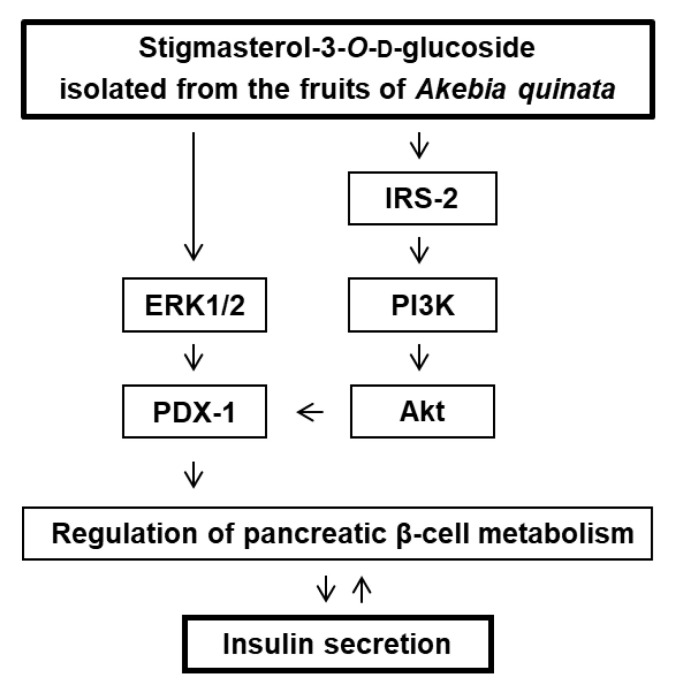
Schematic illustration of the effects of stigmasterol-3-*O*-β-d-glucoside (1) isolated from the fruits of *Akebia quinata* on the expression levels of ERK1/2, IRS-2, PI3K, Akt (Ser473), and PDX-1 proteins in INS-1 cells.

Western blotting was performed for quantifying the expression of the proteins related to mechanisms upstream of PDX-1, following treatment with stigmasterol-3-*O*-β-d-glucoside (1). As depicted in Figure 6A, the expression levels of P-ERK1/2, ERK1/2, P-IRS-2, P-PI3K, and P-Akt (Ser473) proteins markedly increased following treatment with stigmasterol-3-*O*-β-d-glucoside (1) at concentrations of 5 and 10 μM. The bar graphs depict the ratio of P-ERK1/2, ERK1/2, P-IRS-2, P-PI3K, and P-Akt (Ser473) expression normalized to that of GAPDH (Figure 6B–E). The mechanism is summarized by a schematic diagram in Figure 7.

## 3. Discussion

Apart from reducing insulin resistance or increasing the hepatic glucose output, which promotes GSIS, the maintenance of pancreatic β cell function is also an important factor in the treatment of T2DM [32]. In the present study, the exposure of INS-1 cells to AKQU increased the GSIS without inducing cytotoxicity. Chemical analysis of AKQU led to the isolation of stigmasterol-3-*O*-β-d-glucoside (1), maslinic acid (2), scutellaric acid (3), hederagenin (4), akebia saponin PA (5), hederacoside C (6), and hederacolchiside F (7). Hederacoside C (6) and hederacolchiside F (7) weakly increased the GSIS without inducing cytotoxicity. Stigmasterol-3-*O*-d-glucoside (1), maslinic acid (2), scutellaric acid (3), hederagenin (4), and akebia saponin PA (5) increased the GSI by more than four folds without inducing cytotoxicity. Although further study is required, the effect of AKQU on GSIS in INS-1 cells may be the result of synergistic interactions between the compounds 1–7 and the inhibitory interactions by other constituents of the AKQU.

Interestingly, the insulin secretion induced by stigmasterol-3-*O*-β-d-glucoside (1) was similar to that of the anti-diabetic sulfonylurea drug, gliclazide, at a concentration of 10 µM. Maslinic acid (2), a pentacyclic triterpenoid isolated from the fruit of olive trees, has been reported to have anti-inflammatory and anti-oxidant properties [33,34,35]. It has also been shown to have hypoglycemic effects, mediated via the regulation of hepatic glycogen metabolism, in diabetic mice [36,37,38]. Hederagenin (4), a pentacyclic triterpenoid, is present in several plants and possesses various pharmacological properties, including anti-inflammatory, anti-viral, and anti-hyperlipidemic activities [39]. Furthermore, hederagenin (4) possesses anti-diabetic activity mediated via the inhibition of the glycogen phosphorylase enzyme [40]. It has been demonstrated that hederagenin (4) reduces the serum glucose levels in STZ-induced models of diabetes [41]. The results of these previous studies and our present study indicate that maslinic acid (2) and hederagenin (4) may serve as potential natural therapeutic agents for the treatment of T2DM.

There is a paper that analyzes the major chemical constituents of an extract from the fruits of *A. quinata* using the HPLC-ESI-Q-TOF mass spectrometry, and 19 kinds of triterpenoidal saponins including compounds 6 and 7 were identified [42]. Of these, 14 saponins possessed more than four pendent sugars, others were two or three. Stigmasterol-3-O-β-D-glucoside (1), triterpenoids (2-4), and a triterpenoidal saponin with one sugar moiety (5) were active in this study whereas triterpenoidal saponins with five or six sugar moieties (6 and 7) were not. As the number of compounds used in the experiment is limited, it is difficult to predict whether triterpenoidal saponins with two or three sugars such as asperosaponin VI will increase the GSIS or not. Therefore, the correlation between the number of sugars in triterpenoidal saponins and the effect on the GSIS should be studied in the future.

Gliclazide stimulates insulin secretion in patients with T2DM by selectively binding to sulfonylurea receptors on the cell membrane of pancreatic β-cells, and was used as the positive control in this study. It has been additionally reported that gliclazide may have a direct effect on intracellular calcium transport [43]. In this study, we speculated that the mechanism of action of stigmasterol-3-*O*-β-d-glucoside (1) was different from that of gliclazide. Salidroside, isolated from *Rhodiola rosea* (Golden Root), enhances GSIS and improves the nuclear localization of PDX-1 in diabetic db/db mice [44]. A multi-parameter, high-content screening study reported that seven extracts, from a library of 1319 marine invertebrate extracts, reproducibly altered the promoter activity of insulin and/or PDX-1 in murine Min6 insulinoma pancreatic β-cells. The authors finally identified bivittoside D, a lanostane triterpenoid isolated from a sea cucumber collected in Pohnpei, that positively regulated the expression of the insulin gene. Interestingly, the fractions isolated from a sponge collected in Papua New Guinea showed inhibitory effects on the promoter activity of insulin and PDX-1 genes, survival of pancreatic β-cells, and chronic insulin secretion from Min6 cells [45]. PDX-1 plays a critical role in the transcription of the insulin gene and controls the survival and function of pancreatic β-cells [46]. As the critical determinant of T2DM is the loss or dysfunction of insulin-producing pancreatic β cells [47], agents capable of inducing the overexpression of PDX-1 could conceivably cure T2DM.

In the present study, stigmasterol-3-*O*-β-d-glucoside (1) induced the overexpression of PDX-1, while the other four compounds proved to be ineffective. The effect of stigmasterol-3-*O*-β-d-glucoside (1) was attributed to the increase in ERK1/2 phosphorylation. It has been demonstrated that ERK1/2 is a proline-directed Ser/Thr kinase that upregulates the expression of PDX-1, which has three proline-directed Ser/Thr residues [48,49]. Moreover, treatment with stigmasterol-3-*O*-β-d-glucoside (1) increased the phosphorylation of IRS-2 at Ser731, which can potentially activate the PI3K/Akt signaling pathway. The IRS-2 signaling pathway is one of the major signaling pathways that promote the survival of pancreatic β-cells and maintain appropriate β-cell mass [50]. In our previous study, treatment with extracts of *Rehmanniae radix*, *Ginseng radix*, and *Scutellariae radix* increased the GSIS, and this effect was mediated via the induction of IRS2 and PDX-1 in pancreatic β-cell islets isolated from rats [51]. In this study, we observed that treatment with stigmasterol-3-*O*-β-d-glucoside (1) enhanced the PI3K-dependent phosphorylation of Akt at Ser473. The PI3K-dependent phosphorylation of Akt induces the movement of PDX-1 from the nucleus to the cytoplasm and regulates the proliferation of pancreatic β-cells [52,53]. Additionally, PI3K is present upstream of Akt, and is an important factor for the glucose-stimulated transcription of the preproinsulin gene [54]. In consequence, ERK1/2, IRS-2, PI3K, Akt, and PDX-1 could be the major targets responsible for the activity of stigmasterol-3-*O*-β-d-glucoside (1) in promoting insulin secretion in INS-1 cells.

## 4. Materials and Methods

### 4.1. General Experimental Procedures

Column chromatography (CC) was performed on a silica gel (70–230 and 230–400 mesh ASTM, Merck, Kenilworth, NJ, USA), Sephadex LH-20 (GE Healthcare, Chicago, IL, USA), and Diaion HP-20 (Mitsubishi Chemical Co., Tokyo, Japan). High-performance liquid chromatography (HPLC) was performed using the Gilson purification system, with a YMC ODS-A column (250 × 20.0 mm i.d., 5.0 μm, YMC Co., Tokyo, Japan). Thin-layer chromatography (TLC) was performed on silica gel 60 F_254_ and RP-18 F_254S_ plates (Merck). The nuclear magnetic resonance (NMR) spectra were obtained using a JEOL 500 MHz spectrometer, using tetramethylsilane as the internal standard, and the chemical shifts were expressed as δ values. The organic solvents that were used for the chromatographic separations and extractions were distilled before use.

### 4.2. Plant Material

The fruits of *A. quinata* (Thunb.) Decne. (Lardizabalaceae) were purchased from the domestic Korean market (Kyungdong Crude Drugs Market, Seoul, South Korea) in May 2015. The origin of the plant material was identified by one of the authors, D.S.J., and a representative specimen (AKQU-2015) was deposited at the Laboratory of Natural Product Medicine, College of Pharmacy, Kyung Hee University, Republic of Korea.

### 4.3. Extraction and Isolation

The dried fruits of *A. quinata* (2.4 kg) were extracted with 70% aqueous EtOH twice and subsequently evaporated to produce a 70% EtOH extract. The EtOH extract (414.5 g) was suspended in 8 L water and partitioned with ethyl acetate (EtOAc).

The soluble layer of EtOAc (65.4 g) was separated by CC using silica gel (230–400 mesh) as the stationary phase, and subsequently eluted with *n*-hexane/EtOAc/MeOH (9:0.9:0.1), *n*-hexane/EtOAc/MeOH (5:4:1 *v*/*v*), EtOAc/MeOH/water (8.5:1.2:0.3 *v*/*v*), EtOAc/MeOH/water (7:2.5:0.5 *v*/*v*), and 100% MeOH, to obtain 23 fractions (E1–E23). The E16 fraction (408.8 mg) was further fractionated by CC using Sephadex LH-20 and eluted with CH_2_Cl_2_/MeOH (1:1 *v*/*v*) to yield five subfractions (E16-1–E16-5). Scutellaric acid (3, 70.3 mg) was isolated from the E16-3 fraction (95.2 mg), by CC using an ODS gel and eluted with MeOH/water (from 8:2 to 10:0 *v*/*v*). The E14 fraction (403.5 mg) was further fractionated by CC using Sephadex LH-20 and eluted with CH_2_Cl_2_/MeOH (1:1 *v*/*v*) to yield four subfractions (E14-1–E14-4). The E14-3 fraction (166.2 mg) was separated by CC with silica gel (230–400 mesh) and eluted with CH_2_Cl_2_/MeOH/water (from 9.5:0.45:0.05 to 9:0.9:0.1 *v*/*v*) for isolating maslinic acid (2, 108.0 mg). The E15 fraction (410.3 mg) was also subjected to further fractionation by CC using Sephadex LH-20, and eluted with CH_2_Cl_2_/MeOH (1:1 *v*/*v*) to obtain three fractions (E15-1–E15-3). The E15-2 fraction (165.5 mg) was subjected to reversed-phase HPLC with a YMC ODS-A column to yield hederagenin (4, 75.0 mg). Furthermore, stigmasterol-3-*O*-β-d-glucoside (1, 87.8 mg) was engendered by recrystallization from fraction E18 (340.0 mg), and akebia saponin PA (5, 131.0 mg) was isolated from fraction E22 (499.3 mg).

The water-soluble layer (330.8 g) was separated by CC using Diaion HP-20, and eluted with acetone/water (from 0:10 to 10:0 *v*/*v*) to obtain 12 fractions (W1–W12). The W7 fraction (5.1 g) was further fractionated by CC with silica gel (70–230 mesh) and eluted with EtOAc/MeOH/water (6:3:1 *v*/*v*) to obtain three fractions (W7-1–W7-3). The W7-2 fraction (2.1 g) was separated into four fractions (W7-2-1–W7-2-4) by CC with silica gel (70–230 mesh) and eluted with a solvent system of EtOAc/MeOH/water (7:2.5:0.5 *v*/*v*). Hederacoside C (6, 125.8 mg) was obtained from the W7-2-3 fraction (1.3 g) by CC with an ODS gel, and eluted with acetone/water (3:7 *v*/*v*). Hederacolchiside F (7, 11.5 mg) was isolated by CC with silica gel, and eluted with a solvent system of CH_2_Cl_2_/MeOH/water (6:3.6:0.4 *v*/*v*) from the W7-3 (2.5 g) fraction.

### 4.4. Cell Culture

The INS-1 pancreatic β-cell line (Biohermes, Shanghai, China), derived from a rat insulinoma, was used in the present study as an insulin-secreting pancreatic β-cell line. The cells were cultured in monolayer in Roswell Park Memorial Institute (RPMI) 1640 medium (Cellgro, Manassas, VA, USA) supplemented with 2 mM L-glutamine, 0.05 mM 2-mercaptoethanol, 11 mM D-glucose, 1% penicillin/streptomycin (Invitrogen Co., Grand Island, NY, USA), 10% fetal bovine serum (FBS), 10 mM HEPES, and 1 mM sodium pyruvate at 37 °C in a humidified atmosphere containing 5% CO_2_.

### 4.5. Ez-Cytox Cell Viability Assay

The effects of AKQU and compounds 1–7 on the viability of INS-1 cells were assessed by an Ez-Cytox cell viability assay kit (Daeil Lab Service Co., Seoul, Korea) prior to performing the GSIS assay. In order to determine the non-toxic dose ranges of AKQU and compounds 1–7, the INS-1 cells were seeded at a density of 1.5 × 10^4^ cells/mL in 96-well plates for 24 h and subsequently treated with different concentrations of AKQU and compounds 1–7 for 24 h. Thereafter, 10% (*v*/*v*) Ez-Cytox reagent was added to each well and the cells were incubated for 2 h. The optical densities (OD) of each well were measured at 450 nm using a microplate reader (PowerWave XS, Bio-Tek Instruments, Winooski, VT, USA).

### 4.6. LDH Release Assay

The effects of AKQU and compounds 1–7 on the LDH activity in INS-1 cells were assessed by an LDH release assay kit (DOGEN Co., Seoul, Korea) prior to performing the GSIS assay. In order to determine the non-toxic dose ranges of AKQU and compounds 1–7, the INS-1 cells were seeded at a density of 1.5 × 10^4^ cells/mL in 96-well plates for 24 h and subsequently treated with different concentrations of AKQU and compounds 1–7 for 24 h. Thereafter, cell lysates were mixed with LDH assay reagents after solubilizing the cells with 0.5% Triton X-100. The OD of each well were measured at 490 nm using a microplate reader (PowerWave XS, Bio-Tek Instruments, Winooski, VT, USA).

### 4.7. GSIS Assay

The GSIS assay was performed for determining the effects of AKQU and the isolated compounds, 1–7 on insulin secretion in INS-1 cells. In order to determine the GSIS using INS-1 cells, the cells were seeded at a density of 2 × 10^5^ cells/mL in 12-well plates for 24 h. Thereafter, the cells were washed twice with Krebs-Ringer bicarbonate buffer (KRB, pH 7.4) containing 3.3 mM glucose for reproducing the basal condition, and subsequently incubated in fresh KRB containing 3.3 mM glucose for reproducing the starvation conditions. The GSIS of INS-1 cells was measured after 30 min of preincubation with AKQU, compounds 1–7, and gliclazide, followed by 1 h of stimulation with 3.3 mM glucose or 16.7 mM glucose for reproducing basal conditions or for stimulating the INS-1 cells, respectively. The supernatants were collected, and the GSIS was measured using a rat insulin ELISA kit (Gentaur, Shibayagi Co. Ltd., Gunma, Shibukaw, Japan) in accordance to the manufacturer’s instructions. The OD of each condition at 450 nm was measured using a microplate reader (PowerWave XS, Bio-Tek Instruments, Winooski, VT, USA). GSI reflected the GSIS ratio. GSI was measured using the following formula: GSI = insulin level at 3.3 mM glucose/insulin level at 16.7 mM glucose.

### 4.8. Western Blotting

Western blotting was performed for determining whether the expression levels of the proteins related to pancreatic β-cell metabolism were concordant with the effects of AKQU and compounds 1–5 on insulin secretion. The INS-1 cells were seeded at a density of 4.75 × 10^5^ cells/mL in 6-well plates for 24 h and subsequently treated with compounds 1–5 at concentrations of 5 and 10 μM for 24 h. Thereafter, the total protein was extracted for 20 min at 4 °C in RIPA buffer (Cell Signaling, Danvers, MA, USA) containing 1 mM phenylmethylsulfonyl fluoride. The extracts were separated on a 10% sodium dodecyl sulfate polyacrylamide gel, followed by transfer onto polyvinylidene difluoride membranes. The membrane was incubated with the primary antibodies against PDX-1, phospho- ERK1/2 (P-ERK1/2), ERK1/2, phospho-IRS2 (P-IRS2) (Ser731), IRS-2, phospho-PI3K (P-PI3K), PI3K, phospho-Akt (P-Akt) (Ser473), Akt, and glyceraldehyde 3-phosphate dehydrogenase (GAPDH) for 1 h at room temperature. The membrane was subsequently incubated with horseradish peroxidase (HRP)-conjugated anti-rabbit secondary antibody at room temperature for 1 h. The protein bands were subsequently visualized with ECL Plus Western blotting detection reagents (GE Healthcare, Little Chalfont, UK) and a chemiluminescence system (FUSION Solo, PEQLAB Biotechnologie GmbH, Erlangen, Germany).

### 4.9. Statistical Analysis

All the experiments were performed in triplicate. All the analyses were performed using SPSS Statistics version 19.0 (SPSS Inc., Chicago, IL, USA). The non-parametric comparisons were performed by the Kruskal–Wallis test for analyzing the results. A value of *p* < 0.05 was considered to be statistically significant.

## 5. Conclusions

The present study reported that stigmasterol-3-*O*-β-d-glucoside (1), maslinic acid (2), scutellaric acid (3), hederagenin (4), and akebia saponin PA (5) enhanced GSIS in INS-1 cells. Interestingly, the value of GSI following treatment with stigmasterol-3-*O*-d-glucoside (1) was similar to that after treatment with the same concentration of gliclazide. Additionally, treatment with stigmasterol-3-*O*-β-d-glucoside (1) enhanced the induction of PDX-1 via the ERK1/2 and IRS-2 signaling pathways, which control the survival and function of pancreatic β-cells. To the best of our knowledge, this study is the first to report the anti-diabetic activity of stigmasterol-3-O-β-d-glucoside (1). Although further studies are necessary in the future, our findings highlighted the potential of stigmasterol-3-*O*-β-d-glucoside (1), isolated from *A. quinata*, as an attractive drug candidate for the treatment and prevention of T2DM.
